# Tumour necrosis factor-alpha expression in tumour islets confers a survival advantage in non-small cell lung cancer

**DOI:** 10.1186/1471-2407-10-323

**Published:** 2010-06-23

**Authors:** Chandra M Ohri, Aarti Shikotra, Ruth H Green, David A Waller, Peter Bradding

**Affiliations:** 1 Institute for Lung Health, Glenfield Hospital, Leicester, UK; 2 Department of Infection, Immunity and Inflammation, University of Leicester, UK; 3 Department of Thoracic Surgery, Glenfield Hospital, Leicester, UK

## Abstract

**Background:**

The role of TNFα in cancer is complex with both pro-tumourigenic and anti-tumourigenic roles proposed. We hypothesised that anatomical microlocalisation is critical for its function.

**Methods:**

This study used immunohistochemistry to investigate the expression of TNFα in the tumour islets and stroma with respect to survival in 133 patients with surgically resected NSCLC.

**Results:**

TNFα expression was increased in the tumour islets of patients with above median survival (AMS) compared to those with below median survival (BMS)(p = 0.006), but similar in the stroma of both groups. Increasing tumour islet TNFα density was a favorable independent prognostic indicator (p = 0.048) while stromal TNFα density was an independent predictor of reduced survival (p = 0.007). Patients with high TNFα expression (upper tertile) had a significantly higher 5-year survival compared to patients in the lower tertile (43% versus 22%, p = 0.01). In patients with AMS, 100% of TNFα^+ ^cells were macrophages and mast cells, compared to only 28% in the islets and 50% in the stroma of BMS patients (p < 0.001).

**Conclusions:**

The expression of TNFα in the tumour islets of patients with NSCLC is associated with improved survival suggesting a role in the host anti-tumour immunological response. The expression of TNFα by macrophages and mast cells is critical for this relationship.

## Background

Non-small cell lung cancer (NSCLC) is the world's leading cause of cancer related death. At present, the majority of patients present with advanced stages of disease which are not amenable to curative treatment. Even with the optimal presentation of stage Ia disease, the 5-year survival is just 67% [1] assuming fitness for surgical resection. Currently chemotherapy does not offer cure for patients with NSCLC. Thus, it is vital that new biomarkers of disease and novel therapies are developed. It is now recognised that inflammatory and immune responses play a key role in cancer development and prevention [2,3] and it hoped that manipulation of these may yield novel therapies in the future.

Tumour necrosis factor-alpha (TNFα) is a key and proximal component of many inflammatory pathways. It plays a key role in host defence to a variety of pathogens [4,5], but is also implicated in the promotion of many inflammatory diseases [6,7], including rheumatoid arthritis and inflammatory bowel disease.

The tumour biology of TNFα however is complicated, with evidence of both pro-tumourigenic and anti-tumourigenic activity in animal models [8,9]. Perhaps the best evidence that the predominant role played by TNFα is anti-tumourigenic arises from studies of anti-TNFα strategies for the treatment of inflammatory disease in man. These show a significant increase in the rate of neoplastic disease in patients receiving active treatment, and no evidence of protection against cancer development [10-16]. The complete resolution of NSCLC in a patient following withdrawal of anti-TNFα therapy is also described [17]. In contrast, the administration of anti-TNFα has had no significant effect on the progression of several advanced cancers [18-20]. Furthermore, recombinant TNFα is a useful and licensed adjunctive treatment for sarcoma and melanoma [21,22].

There is therefore debate as to whether or not TNFα plays a role in NSCLC tumour cytotoxicity or conversely, tumour progression. Two small studies investigated previously the mRNA or protein expression of TNFα in NSCLC, and suggested overall that TNFα expression was either mildly beneficial but not an independent factor [23] or neutral [24], respectively. However, the anatomical localisation of the TNFα expressed was not taken into account. We have shown previously that the site of inflammatory cell infiltration in NSCLC is critical in terms of prognosis. Patients with high expression of macrophages in the tumour islets have extended survival independently of tumour stage, and these macrophages demonstrate high expression of TNFα and other cytotoxic markers, suggesting they are of an anti-tumourigenic cytotoxic M1 macrophage phenotype [25,26].

This previous work investigating TNFα expression in macrophages was aimed primarily at determining the phenotype of these cells rather than the prognostic significance of TNFα expression. Due to the nature of the work, relatively small numbers of patients were studied. The aim of this study was therefore to assess the prognostic significance of TNFα expression, irrespective of cell type in NSCLC, paying particular attention to its anatomical microlocalisation, in surgically resected NSCLC in our complete cohort of patients described previously [26].

## Methods

### Study Population

The study was approved by the Leicestershire Research Ethics Committee. The tissue specimens evaluated were from patients with NSCLC who had undergone resection with curative intent at the University Hospitals of Leicester National Health Service Trust (Leicester, United Kingdom). These patients had resections during two periods - one dating from 1991 to 1994 and the second from January to December 1999. This cohort of patients has been described previously [26]. Of note, due to exhaustion of tumour tissue, 133 patient samples with >60 day survival post surgery were available for analysis in this study. Of the 133 patients studied, 88 were men and average age at surgery was 65.8 years (standard deviation, 9.8; range, 33 to 82 years). Full clinicopathologic information was gathered before and after surgery, including patient characteristics, treatment, combined clinical and surgical staging results (preoperative staging by computed tomography scan, selective mediastinoscopy, and systematic lymph node sampling at operation), histologic subtype, tumour grade, and survival data. Patients were divided into two groups: above median survival (AMS) (mean ± SEM 84.0 ± 5.1 months) and below median survival (BMS) (mean ± SEM 10.8 ± 0.8 months). Macrophage-associated TNFα expression has been described previously in 26 of these patients [25]. Patient characteristics are shown in Table 1.

**Table 1:** Patient Characteristics.

Characteristic	Extended Survival	Poor Survival
No. of patients	67	66
		
Age - years	66.3 ± 1.2	65.3 ± 1.2
		
Male sex - no. (%)	39 (58)	49 (74)
		
Year of surgery - no. (%)		
1991	0 (0)	5 (7)
1992	5 (7)	6 (9)
1993	6 (9)	4 (6)
1994	6 (9)	7 (11)
1999	50 (75)	44 (67)
		
Tumour stage - no. (%)		
1	40 (60)	20 (30)
2	18 (27)	19 (29)
3a	9 (13)	23 (35)
3b and 4	0 (0)	4 (6)
		
Histology - no. (%)		
Squamous	39 (58)	29 (44)
Adenocarcinoma	19 (28)	23 (35)
Large cell	4 (6)	8 (12)
Other	5 (7)	6 (9)
		
Tumour Grade - no. (%)		
Well	5 (7)	2 (3)
Moderate	31 (46)	17 (26)
Poor	30 (45)	46 (70)
Not recorded	1 (1)	1 (1)
		
Adjuvant Chemotherapy (%)	0 (0)	2 (3)
		
Radiotherapy (%)	8 (12)	12 (18)
Palliative Radiotherapy (%)	7 (10)	9 (14)
Survival - months	84.0 ± 5.1	10.8 ± 0.8

Plus-minus values are means ± SEM

### Immunohistology

Specimens studied were formalin fixed and paraffin embedded. Only blocks containing the advancing edge of the primary tumour were evaluated. Tissue sections of 4 *μ*m thickness were cut onto glass slides and then de-waxed in xylene and rehydrated through graded alcohols. Antigen retrieval was carried out using Trilogy Antigen Retrieval solution (Cell Marque, Hot Springs, United States of America) in a pressure cooker (heated to 117.5°C for 1 min and then cooled to 100°C for 30 seconds). TNFα mouse antihuman antibody was used (clone P/T2; Abcam, Cambridge, United Kingdom). Immunostaining was performed and TNFα was developed with peroxidase and 3,3'-diaminobenzidine tetrahydrochloride (brown reaction product). Sections were then counterstained with haematoxylin and mounted in an aqueous mounting medium (BDH Chemicals Ltd, Poole, United Kingdom). Appropriate isotype controls were performed where the primary antibodies were replaced by irrelevant mouse mAb of the same isotype and at the same concentration as the specific primary mAb. To assess whether TNFα + cells were localised to mast cells, a double-stain technique described previously by us was used [25,26], with the mouse monoclonal antibody to human tryptase (clone AA1, Dako Cytomation, Ely, Cambridgeshire, United Kingdom) employed to identify mast cells.

### Analysis and Validation of Immunostaining

Analysis was performed blind with respect to the clinical outcome. The ten most representative high-power fields (x400) per slide were manually selected using an Olympus BX50 microscope (Olympus, Southall, United Kingdom). The respective areas of stroma and tumour islets were then measured at x400 magnification using Analysis imaging software (Soft Imaging System GmbH). The number of nucleated cells with positive staining for TNFα in each area were then counted manually and expressed as cells/mm^2 ^of stroma or tumour islets. Analysis was repeated for 20 patients to assess repeatability and validity.

### Statistical Analysis

Statistical analyses were carried out using the GraphPad Prism software package (v. 4.02; GraphPad Prism Software Inc, San Diego, CA). For categoric analysis, the median value was used as a cut point to dichotomise the series. The χ^2 ^test was used to test for relationships between categoric variables, and the Mann-Whitney nonparametric test was used to compare categoric with continuous variables. The Kruskal-Wallis one-way analysis of variance test was used to compare multiple groups. Kaplan-Meier survival curves were used to look for correlations with survival and were compared with the use of the log-rank statistic. For the above comparisons, p < 0.05 was considered statistically significant. A multivariate Cox proportional hazards model was used to estimate adjusted hazard ratios, 95% CIs, and to identify which of the macrophage markers were independent prognostic factors using the Statistical Package for the Social Sciences (SPSS, v. 13.0; SPSS Inc, Chicago, IL). The validity of the proportional hazards assumption was assessed from log(-log [Survival]) curves.

## Results

### Patient Characteristics

Patent characteristics are shown in Table 1. Of the 133 patients studied, 110 had died at the time of analysis. Thirty-day mortality for the cohort was 4% and patients who died within the first 60 days of surgery were not included in this analysis. Sixty-eight tumours were squamous, 42 adenocarcinoma, 12 large cell, and 11 other. Sixty were stage I, 37 stage II, 32 stage IIIa, and four stage IIIb or IV. Two patients had additional postoperative chemotherapy and 20 had additional radiotherapy, 16 of whom had it for later palliation. Neither had any effect on survival. The overall 5-year survival was 31.3%.

### Validation of Analysis

**Figure 1:**
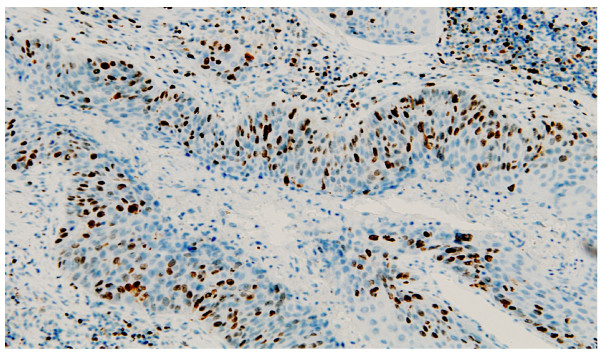
**Immunohistology demonstrating positive TNFα expression (brown)**. Magnification ×100.

Clear and distinguishable staining was evident for TNFα (Figure 1). Appropriate isotype controls were negative. In order to assess the validity of the method, area measurements and cell counts were repeated and intraclass correlation coefficients calculated. Good correlations were found for both: 0.997 (95% CI, 0.996 to 0.998, p < 0.001) and 0.994 (95% CI, 0.992 to 0.996, p < 0.001). This method of analysis has also been validated by our group previously [26].

### Cellular Distribution

**Figure 2:**
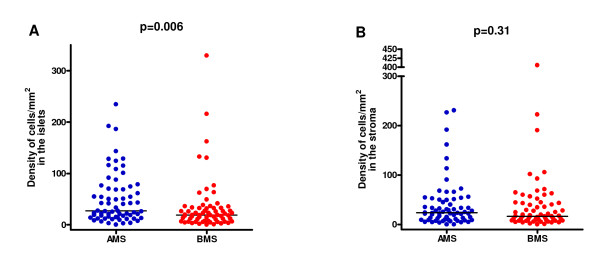
**TNFα densities in Above Median Survival (AMS) and Below Median Survival (BMS) patients in the tumour islets (A) and stroma (B)**.

There was increased expression of TNFα in the tumour islets of patients with AMS compared to those with BMS (median 27.2 versus 18.8 cells/mm^2 ^respectively, p = 0.006). There was no significant difference in the expression of stromal TNFα between the two groups (AMS 23.8 versus BMS 16.8 cells/mm^2^, p = 0.31) (Figures 2A and 2B).

### Clinical Outcome

**Figure 3:**
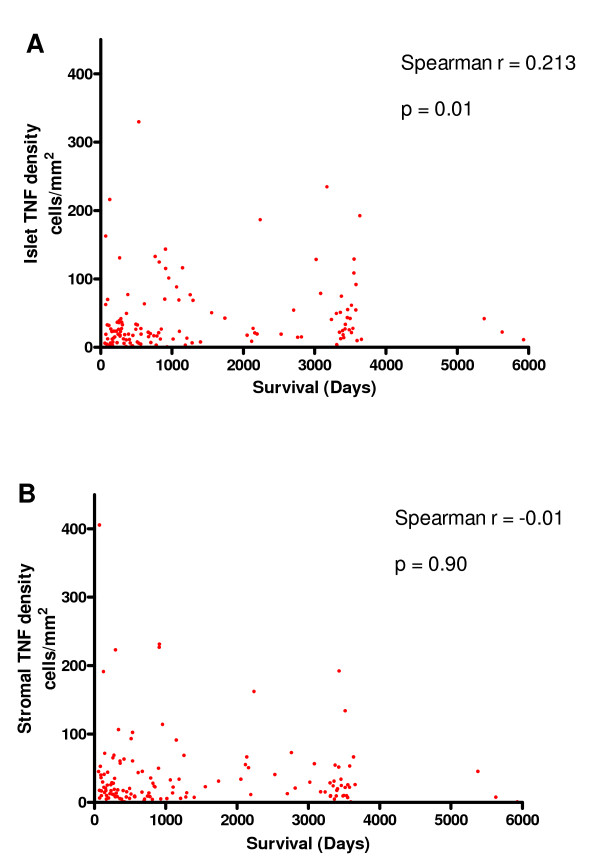
**Raw data of cell counts expressing TNFα plotted against survival in days in the tumour islets (A) and stroma (B)**.

Scatter plots of the raw data of TNFα density versus survival are shown in Figure 3. Spearman's rank correlation coefficient was calculated to assess any potential relationship with survival. A direct relationship between tumour islet TNFα density and survival was noted (r_s _= 0.213, p = 0.01). No significant relationship was seen between survival and stromal TNFα density (r_s _= -0.01, p = 0.90).

### Kaplan-Meier Survival Analysis

**Figure 4:**
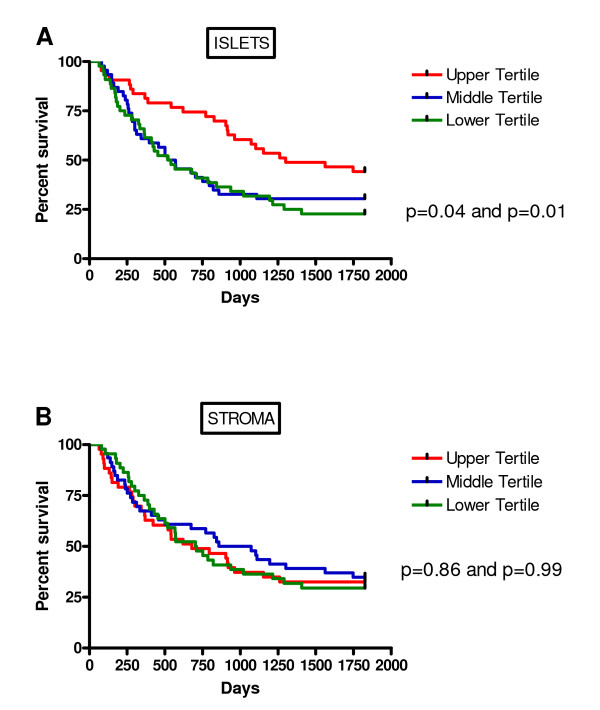
**Kaplan-Meier five year survival curve for TNFα densities in the tumour islets (A) and stroma (B) divided into high counts (upper tertile), midrange counts (middle tertile) and low counts (lower tertile)**. The p values in (A) and (B) reflect the difference between the upper tertile and middle tertile groups and also the difference between the upper and lower tertile groups.

**Figure 5:**
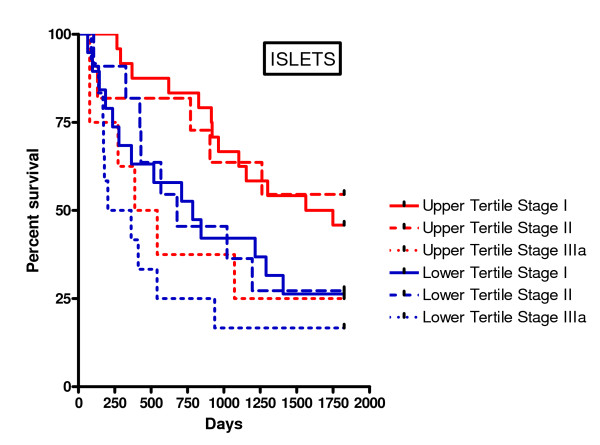
**Kaplan-Meier five year survival curve for TNFα densities in the tumour islets for each tumour stage using upper and lower tertile values for TNFα density**. For comparison of upper tertile stage I versus lower tertile stage I p = 0.06, for upper tertile stage II versus lower tertile stage II p = 0.18, for upper tertile stage IIIa versus lower tertile stage IIIa p = 0.55, and for upper tertile stage IIIa versus lower tertile stage I p = 0.70.

For further analysis, the data were divided into two or three equal groups according to TNFα density. Kaplan-Meier survival curves were plotted and the log-rank statistic used to compare survival rates. When looking at two groups separated by the median, there was a non-significant trend for improved survival with above median TNFα expression in the tumour islets (p = 0.15, not shown). When divided into tertiles, there was significantly improved survival in the top tertile of TNFα expression in the tumour islets compared to the middle and lower tertiles (Figure 4A). Thus patients with high islet TNFα expression (upper tertile) were noted to have a significantly higher 5-year predicted survival as opposed to patients with low TNFα expression (lower tertile) (43% versus 22%, p = 0.01). There was no significant relationship between TNFα expression in the stroma and survival (Figure 4B). Similar differences in survival with respect to TNFα expression in the tumour islets were also evident within tumour stages (Figure 5), although the differences did not reach statistical significance due to the smaller numbers within each stage. Interestingly, patients with stage IIIa disease in the top tertile of islet TNFα expression had a 5 year survival of 25% compared to 26% survival for those patients with stage I disease in the lower tertile of islet TNFα expression.

### Multivariate Cox Proportional Hazards Analysis

A Multivariate Cox Proportional Hazards model was used to estimate adjusted hazard ratios, 95% CIs, and to assess whether TNFα islet or stromal counts were independent prognostic factors, using a forward stepwise method with a probability of 0.05 for entry and 0.10 for removal by likelihood ratio statistics. Results of the multivariate analysis are shown in Table 2. Continuous variables were used for islet and stromal TNFα. For every increase of 1 in the value of the variable, the hazard increases by the value of the hazard ratio. Expression of TNFα in the tumour islets emerged as a significant independent predictor of survival (hazard ratio 0.995, 95% CI 0.989 to 1.000, p = 0.048). Surprisingly, expression of TNFα in the tumour stroma emerged as a significant independent predictor of reduced survival (hazard ratio 1.007, 95% CI 1.002 to 1.011, p = 0.007). There was no evidence of violation of the proportional hazards assumption.

**Table 2:** Results of Cox Regression Analysis.

Factor	Hazard Ratio	95% CI	p
Islet TNFα	0.995*	0.989 to 1.000	0.048†
			
Stromal TNFα	1.006*	1.002 to 1.011	0.007†
			
Age	1.022	1.000 to 1.043	0.049†
			
Pathological stage			0.002†
I	1.000		
II	0.283‡	0.077 to 1.035	0.056
IIIa	0.373	0.102 to 1.372	0.138
IIIb and IV	0.712	0.194 to 2.619	0.609
			
Grade - differentiated			0.078†
Well	1.000		
Moderate	0.481§	0.177 to 1.306	0.151
Poor	0.752	0.488 to 1.159	0.197
			
Histology			0.711
Squamous	1.000		
Adenocarcinoma	0.708	0.323 to 1.553	0.389
Large	0.828	0.363 to 1.891	0.654
Other NSCLC	0.621	0.235 to 1.640	0.336

*Continuous variable used. For every increase of 1 in the value of the variable, the hazard increases by the value of the hazard ratio.

† Overall significance as a prognostic factor

‡ Hazard relative to stage I

§ Hazard relative to well differentiated

¶ Hazard relative to squamous

### Cellular localisation of TNFα

**Figure 6:**
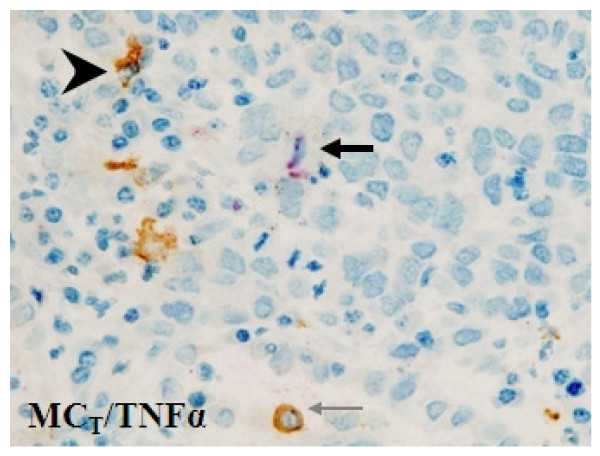
**Mast cell immunohistochemical double-staining tryptase (brown) and TNFα (red) demonstrating the presence of TNFα in tryptase + mast cells**. Arrowhead = double-stain cell. Black arrow = single-stain red cell. Grey arrow = single-stain brown cell.

We have shown previously that in patients with extended survival, there is increased expression of macrophages in the tumour islets compared to patients with poor survival [26], and that in a separate study the majority of tumour islet macrophages expressed TNFα [25]. Macrophages accounted for approximately 60% of the TNFα + cells in the extended survival patients. While there was a marked difference in the number of TNFα + macrophages between extended survival and poor survival islets [25], it is evident from Figure 2 that the difference in total TNFα expression in this cohort of 133 patients is not so marked. Based on morphology, it was considered that a proportion of the TNFα + cells that were not macrophages may be mast cells. We therefore analysed the cellular distribution of TNFα expressed by mast cells (Figure 6) in the subset of samples previously stained for macrophage-TNFα (AMS [n = 20] and BMS [n = 20]) [25]. In keeping with our previous observations [26], mast cell numbers were significantly increased in the islets of the AMS compared to BMS patients (medians 22.6 versus 0.7 cells/mm^2^, p < 0.001). Interestingly, mast cells accounted for 45.2% of TNFα expression in the islets of the AMS patients, but only 5.2% of the BMS patients (Table 3)(p < 0.001). Thus taking macrophage and mast cell-associated TNFα together, approximately 100% of islet TNFα was localized to mast cells or macrophages in the subset of AMS patients studied, while in the islets of the subset of BMS patients studied, only 28% of TNFα immunoreactivity was localized to these cells (Table 3) (p < 0.001). Similar results were evident in the tumour stroma (Table 3, p < 0.001). This is interesting because it demonstrates that in BMS patients, there is robust TNFα expression by cells other than macrophages and mast cells. These cells were predominantly mononuclear cells and rarely tumour epithelial cells. This suggests that TNFα is highly beneficial only when localized to macrophages and mast cells in tumour islets, and not when expressed by other cell types.

**Table 3:** Assessment of the percentage of cell types expressing TNFα in patients with NSCLC in above median survival patients in the islets (AMSI) and stroma (AMSS) and below median survival patients in the islets (BMSI) and stroma (BMSS).

	AMSI	AMSS	BMSI	BMSS
% of cells which were Macrophages	61.8* (9.4-100)	76.9† (16.6-100)	22.2 (5-71.5)	39.7 (0-61.9)
% of cells which were mast cells	45.2# (20.3-58.4)	54.9¶ (0-79.1)	5.6 (0-100)	10.5 (0-55)
Estimated % of other cell types	0	0	72	50

*p < 0.001 compared to BMSI; †p < 0.001 compared to BMSS; #p < 0.001 compared to BMSI; ¶p < 0.001 compared to BMSS. We analysed the cellular distribution of TNFα expressed by mast cells in the subset of samples previously stained for macrophage-TNFα [25].

## Discussion and Conclusions

The purpose of this study was to investigate the relationship between the microanatomical expression of TNFα and survival in surgically resected NSCLC. The results demonstrate that expression of TNFα in the tumour islets is associated with a significant increase in 5-year survival, independently of other favorable prognostic factors including stage, and that TNFα expression in the stroma is an independent predictor of reduced survival.

The role of TNFα in tumour biology remains controversial with both pro-tumourigenic and anti-tumourigenic properties identified [8-10,21,27]. Two small previous studies of patients with NSCLC suggested TNFα expression had little relationship to clinical outcome, but these did not distinguish between expression in tumour stroma and epithelial islets. The biology of these two tumour compartments demonstrates profound differences in matrix composition, cellular content and vascularity. Our previous studies [25,26] have demonstrated the importance of anatomical microlocalisation in terms of potential sites for cytotoxicity against tumours. The key findings of these studies was that tumour epithelial islets are the likely site of host cytotoxic responses against tumour progression because patients with extended survival have infiltration of their tumour islets with mast cells and macrophages [25,26]. Many cytokine-dependent effects are mediated through localized cell-cell contact, including the presentation of membrane-bound TNFα to TNFα-receptor + cells [28]. The effects of cytokines including TNFα on tumour stroma versus islets are therefore likely to vary profoundly depending on the site of release, and the cell-cell interactions between TNFα-producing cells and the cells they interact with. We therefore analysed TNFα expression with close attention to expression in the stroma versus the tumour islets.

In keeping with biological relevance of this concept, patients with AMS survival had greater immunoreactive TNFα expression in their tumour islets compared to those with BMS, and there was a positive correlation between increasing islet TNFα + cell density and survival. In addition, when our patient cohort was divided into tertiles according to islet TNFα + cell density we observed that patients with high expression (upper tertile) had significantly improved 5-year survival. Thus increasing expression of TNFα in the tumour islets was associated with a significant increase in 5-year survival, independently of other favorable prognostic factors. Conversely, although the density of TNFα + cells in the stroma was similar in AMS and BMS patients, TNFα expression in the stroma emerged as an independent predictor of reduced survival following Cox regression analysis. These results indicate that the micro-anatomical location of TNFα expression is potentially critical in determining its role in tumour biology.

Tumour stage is a key determinant of survival following surgery for NSCLC [1]. We were able to show that the relationship between survival and islet TNFα expression was evident within tumour stages although due to the relatively small numbers for each stage the differences did not reach statistical significance. Patients with stage I disease survive longer than patients with stage IIIa disease [1]. However, when tumour islet TNFα expression was compared by stage with respect to survival, it was of note that patients with stage IIIa NSCLC and high TNFα expression had a 5-year survival of 25%, comparable to that of stage I NSCLC patients with low TNFα expression, who had a 5-year survival of 26%. Conversely, patients with stage I NSCLC and high TNFα expression have a 5-year survival of 46%. This suggests that TNFα expression within tumour islets is therefore a key determinant of NSCLC survival within stage, even within early stage disease.

A striking observation from this study was the cellular distribution of TNFα immunoreactivity in the tumour islets of patients with extended survival compared to those with poor survival. The macrophages which infiltrate the tumour islets in NSCLC are predominantly of the M1 cytotoxic phenotype [25] which express TNFα. We have extended this work in the current study and show that mast cells within the tumour islets also express TNFα. What is striking, however, is that in the subset of patients with extended survival, all TNFα islet immunoreactivity was accounted for by macrophages and mast cells. In the subset of patients with poor survival, TNFα expression in the islets was also evident, but was rarely present in mast cells or macrophages. Thus it is not only expression of TNFα in the tumour islets that is critical in determining survival, but perhaps more importantly, the type of cells which are expressing it.

The distinction of tumour islets versus stroma is important as there is evidence suggesting that TNFα located in the stroma contributes towards tumour proliferation via angiogenesis [29]. In support of this, although there was no difference in the stromal density of TNFα + cells in AMS versus BMS patients, increasing stromal TNFα expression emerged as an independent predictor of worse survival. Whether this is due to the effects of TNFα on the stroma or a reflection of the inability of potentially beneficial TNFα-expressing macrophages and mast cells to infiltrate the islets is not known. The latter could be explained by an inappropriate chemokine repertoire released by the tumour stroma or even by a physical barrier, such as a thickened basement membrane, between the stroma and tumour islets.

The results of this study add to our previous work and have important clinical implications. We have shown that the density of TNFα + cells in tumour islets is a predictor of extended survival in NSCLC following surgery. Its localization to macrophages and mast cells in the tumour islets is the key factor relating to improved prognosis, and it seems unlikely that anti-TNFα strategies will be beneficial to such patients. In contrast, in poor prognosis patients whose tumours contain relatively few TNFα + macrophages and/or mast cells, anti-TNFα strategies may be worthy of further study as stromal expression is an independent predictor of poor survival. In view of the marked microanatomical and immunological heterogeneity within the tumour microenvironment in NSCLC, it is essential that attention is paid to this principle in future immunomodulatory trials in this disease. Targeting subphenotypes of disease with immunopathology predicted to respond to the intervention may then lead to the development of better anti-neoplastic therapeutic strategies.

## Competing interests

The authors declare that they have no competing interests.

## Authors' contributions

CO carried out the immunohistochemical staining, slide analysis, statistical analysis and prepared the manuscript. AS assisted with microtomy and the immunohistochemical staining. RG carried out the statistical analysis. DW provided the samples for analysis and prepared the manuscript. PB conceived the study, participated in its design, coordination and analysis, and prepared the manuscript. All authors read and approved the final manuscript.

## Pre-publication history

The pre-publication history for this paper can be accessed here:

http://www.biomedcentral.com/1471-2407/10/323/prepub
